# Disability prevalence in midlife (aged 55–65 years): Cross-Country comparisons of gender differences and time trends

**DOI:** 10.1186/s40695-020-00061-0

**Published:** 2021-01-02

**Authors:** Serena Wang, Drystan Phillips, Jinkook Lee

**Affiliations:** 1 Krieger School of Arts and Sciences, Johns Hopkins University, MD Baltimore, USA; 2 Center for Economic and Social Research, University of Southern California, CA Los Angeles, USA; 3 Department of Economics, University of Southern California, CA Los Angeles, USA; 4 RAND Corporation, CA Los Angeles, USA

**Keywords:** Functional Limitations, Longitudinal, Global, Activities of Daily Living, Instrumental Activities of Daily Living

## Abstract

**Background:**

Prior literature on disability has centered on disability prevalence among older adults ages 65 and older, providing only limited insight to potential gender differences in disability prevalence in mid-life. Midlife is, however, a critical time to be examined, as it is typically the time in the life course when large inequalities in physical health first emerge.

**Methods:**

Using the Harmonized data files provided by the Gateway to Global Aging Data, we estimate disability prevalence of nationally representative adults ages 55–65 from 23 countries (*N* = 79,465). We examine gender differences in two disability indicators, limitations in instrumental activities of daily living (IADLs) and activities of daily living (ADLs) in two time periods, 2004/05 and 2014/15.

**Results:**

There are substantial cross-country variations in IADL and ADL disability prevalence in midlife. Within countries, we find that women have higher IADL prevalence than men in only one out of five countries. Similarly, for ADL prevalence, women have higher ADL prevalence than men in only one out of ten countries. Further, comparing disability prevalence in two time periods, we observe different country-specific time trends.

**Conclusions:**

In the majority of mid and high-income countries, there is no significant gender difference in IADL and ADL prevalence, but there are few countries where women show higher prevalence of disability than men in mid-life. This finding calls for future research into what contributes to cross-country variations.

## Background

Although women throughout the world have surpassed men in longevity [1], they have been often found to be more vulnerable for disability than men. These patterns of higher disability prevalence for women persist across several continents including North America [2, 3], Europe [4–6] and Asia [7]. As women’s life expectancy has surpassed men’s, these gender differences in disability may reflect the differences in longevity. However, in many studies, even after controlling for age, women are still found to be more vulnerable for disability at older ages compared to men [8–10]. These widespread gender differences among older adults bring into question whether this global pattern of higher disability in women is also present during earlier stages of life.

Literature on disability heavily focuses on older populations, typically 65 years and older, and has consistently found that older women have higher disability prevalence compared to men [2]. Performance on two disability measures are typically assessed: instrumental activities of daily living (IADL) and activities of daily living (ADL). ADL includes skills required to manage basic physical needs, and IADL includes more complex activities associated with the ability to live independently [11, 12]. One recent study, using pooled data from the World Health Survey covering ages 50 and older in 57 countries, reported that on average the disability prevalence rate is higher among older women than older men, and the gender gap widens with age: for ages 75–79, the disability prevalence rate was 56.3% for women and 44.5% for men, but for ages 80+, the disability rate was 68.9% for women and 45.5% for men [13]. Similar patterns have been observed in country-specific studies in the United States [9], Canada [14], and Europe among ages 70+ [2] and in cross-country studies of the U.S. and Europe among age 70+ [2] and of Denmark, Japan, and the U.S. among age 85+ [15].

Unlike for older populations, an empirical consensus is lacking for disability prevalence in midlife, despite a growing recognition that the mid-life period is typically the time when large inequalities in physical health begin [16, 17]. Several studies have found that women experience declines in physical ability earlier than men. For example, in a middle-aged cohort study, British women were found to have weaker handgrip strength and poorer balance time compared to men [18]. Another study found that middle aged women were more likely to report experiencing worse balance and difficulties in stair climbing than middle aged men [19]. Further, it has been noted that the onset of limitations in physical functioning for women often coincides with the timing of menopause [20–22].

Recognizing the critical importance of mid-life, expanded research attention on disability has been paid to the mid-life [23]. Liang and colleagues [24] found a gender gap in combined IADL/ADL disability prevalence in the United States for people ages 55 years in 1995 over an 11-year time period. In contrast, however, Crimmins et al. found no significant gender effect in IADL or ADL difficulties in 2004/2005 for ages 50–59 in the US, England, or a pooled sample of 11 European countries [2]. Looking into potential geographic variations within Europe, Scheel-Hincke et al. [25] found a gender difference in both IADL and ADL disability in Southern Europe, a gender difference only in IADL disability in Northern and Western Europe, and no gender difference in disability in Eastern Europe for ages 50–64^1^, using data from 2004 to 2015 SHARE. Pallon et al. [26] considered ADL disability in Mexico for people age 50–59 in 2001 and found no significant gender difference.

In this paper, we aim to investigate disability in mid-life, particularly whether gender disparities exist at ages 55–65. Because there are cross-country variations in gender inequity, we hypothesize such inequity will translate to health disparity, leading to cross-country variations in gender differences in disability in midlife. Furthermore, because the reduction of gender disparities has evolved differently across countries, we expect gender differences in disability to also vary among countries over time. To study disability at mid-life between genders and across different countries and times, we utilize Harmonized data from the Gateway to Global Aging Data to analyze gender differences in disability prevalence using two measures of disability for 23 countries. These countries include Austria, Belgium, China, Croatia, Czechia, Denmark, England, Estonia, France, Germany, Greece, Israel, Italy, Korea (South), Luxembourg, Mexico, Poland, Portugal, Slovenia, Spain, Sweden, Switzerland, and the United States.

## Methods

We use Harmonized data files provided by the Gateway to Global Aging Data (Gateway), which are easy-to-use individual-level longitudinal data constructed for cross-wave and cross-country analyses. Currently, the Gateway offers ten Harmonized datasets with 23,200 key harmonized variables on demographics, health, financial and housing wealth, income, family structure, retirement, employment history, cognition, consumption, health care, pensions, and stress. The Harmonized data file are built from original data collected as part of the Health and Retirement Study (HRS) and its sister studies around the world. Started in 1992, HRS is a panel study that surveys a representative sample of Americans over the age of 51 and their spouses of any age. Following the success of the HRS, several sister studies have been designed, modeling after the HRS, including the Mexican Health and Aging Study (MHAS), English Longitudinal Study of Ageing (ELSA), Korean Longitudinal Study of Aging (KLoSA), Survey of Health, Ageing, and Retirement in Europe (SHARE), and Chinese Health and Retirement Longitudinal Study (CHARLS). Enabling cross-country comparisons is an important goal of the HRS family of survey, and most of the surveys share the following characteristics: (i) biennial interviews with respondents and their spouses regardless of age; (ii) a multidisciplinary questionnaire design providing wealth of information about health, socioeconomic status, demographics, and other topics; and (iii) regular refreshment samples to keep the sample representative of the older population.^2^

The files used in this paper include: the RAND HRS Longitudinal File 2016 (V1) [27], Harmonized MHAS Version A [28], Harmonized ELSA Version F [29], Harmonized SHARE Version D.5 [30], Harmonized KLoSA Version C [31], and Harmonized CHARLES Version C [32]. For this analysis we focus on interviews conducted during two time points, in 2004/05 and 2014/15. A few countries, Czechia, South Korea, Mexico, and Poland did not conduct a survey in 2004/05, for these countries data from the closest available survey year were used, provided it was not more than 2 years outside of our target time point. The details of interview years and sample sizes and mean age by gender are summarized in Table 1. There are no statistically significant differences in mean age between genders inside each country/time point sample except for the 2014/2015 samples in England and Greece where the difference is less than a single year.

**Table 1:** Sample descriptions by country

		2004/2005 Respondents Age 55 - 65							2014/2015 Respondents Age 55 - 65						
			Men			Women				Men			Women		
Country	Survey	Interview year(s)	N	Mean age	SD age	N	Mean age	SD age	Interview year(s)	N	Mean age	SD age	N	Mean age	SD age
Austria	SHARE	2004	284	60.39	3.10	369	60.05	3.28	2015	505	60.30	3.34	666	60.33	3.15
Belgium	SHARE	2004, 2005, 2006	650	59.30	3.09	708	59.49	3.18	2015	1,000	60.05	3.17	1,154	59.78	3.14
China	CHARLS								2015, 2016	3,566	60.19	3.04	3,740	60.20	3.04
Croatia	SHARE								2015	500	60.33	3.16	583	59.90	3.11
Czechia	SHARE	2006, 2007	513	59.73	3.10	665	59.80	3.10	2015	745	60.79	2.99	1,072	60.45	3.05
Denmark	SHARE	2004	293	59.47	3.04	323	59.39	2.97	2015	673	59.91	3.15	760	59.83	3.13
England	ELSA	2004, 2005	1,692	59.61	3.11	1,969	59.52	3.10	2014, 2015	1,540	60.77	2.91	1,886	60.42	2.95
Estonia	SHARE								2015	776	60.22	3.12	1,042	60.13	3.15
France	SHARE	2004, 2005	489	59.32	3.18	573	59.50	3.21	2015	661	60.36	3.09	769	60.22	3.09
Germany	SHARE	2004	553	60.42	3.14	591	60.37	3.20	2015	755	60.37	3.19	911	60.31	3.16
Greece	SHARE	2004, 2005	473	59.66	3.16	467	59.65	3.19	2015	760	60.80	3.12	1,045	60.27	3.15
Israel	SHARE	2005, 2006	405	59.50	3.08	524	59.27	2.99	2015	270	61.01	2.68	415	60.82	2.97
Italy	SHARE	2004	502	60.02	3.14	626	60.04	3.23	2015	798	60.43	3.13	1,000	60.00	3.16
Korea	KLoSA	2006	1416	60.05	3.16	1,690	60.13	3.22	2014	1,203	59.71	3.21	1,425	59.81	3.18
Luxembourg	SHARE								2015	302	59.81	3.13	393	59.58	3.09
Mexico	MHAS	2003	2,635	59.48	3.13	3,110	59.53	3.11	2015	1,856	60.02	3.33	3,018	60.10	3.12
Poland	SHARE	2006, 2007	427	59.58	3.19	518	59.25	2.98	2015	347	60.52	3.01	423	60.26	2.95
Portugal	SHARE								2015	299	60.04	2.88	380	59.65	3.17
Slovenia	SHARE								2015	755	60.50	2.99	936	60.18	3.02
Spain	SHARE	2004	327	59.96	3.12	422	59.73	3.14	2015	879	60.43	3.11	1,035	60.16	3.17
Sweden	SHARE	2004, 2005	568	59.86	3.08	661	59.71	3.12	2015	501	61.33	2.87	620	61.00	2.95
Switzerland	SHARE	2004	174	59.99	2.91	179	59.90	3.08	2015	461	60.18	3.03	622	59.92	3.15
USA	HRS	2004, 2005	2,665	60.61	3.34	3,624	60.39	3.24	2014, 2015	3,232	59.73	3.12	4,156	59.83	3.15

Notes: [1] For 2004/2005 some interviews were also conducted in 2006 for Belgium, 2006-07 interviews were used for Czechia, some interviews were in 2006 for Israel, 2006 interviews were used for Korea, 2003 interviews were used for Mexico, 2006-07 interviews were used for Poland.

[2] No interviews were collected close to 2004/2005 for China, Croatia, Estonia, Luxembourg, Poland, and Slovenia.

[3] For 2014/2015 some interviews were also conducted in 2016 for China.

[4] Mean age is not derived using weights so as to provide an accurate representation of the sample.

All six studies in our analysis include questions about activities of instrumental daily living (IADLs) [11] and activities of daily living (ADLs) [12, 33]. In general, each study asks respondents whether they have had difficulty with each IADL and ADL activity because of a physical, mental, emotional, or memory problem, excluding difficulties expected to last less than three months. In the HRS and MHAS, in addition to yes and no answers, respondents could also answer that they can’t do or don’t do the IADL or ADL activity. For IADLs, respondents were then asked whether they can’t or don’t do the activity because of a health or memory problem. For ADLs, respondents were then asked whether anyone helps them with that activity. Also in the HRS and MHAS, if the respondent had previously reported no difficulty to any of a dozen other functional limitations (e.g. running a mile), they were not asked ADL questions. The question wording in KLoSA differs as it asks whether respondents can do the IADL/ADL activity by themselves or whether they need help.

We have built harmonized IADL variables and harmonized ADL variables to address the differences in response choices, survey skip pattern, and question wording. In designing harmonized IADL variables we treated cases where the respondent said they don’t do or can’t do the activity because of a health or memory problem as having difficulty. Similarly, we also accounted for don’t do and can’t do responses to ADL questions by treating respondents as having difficulty when we also know that someone helps them with the activity. For harmonized ADL variables, if the respondent was not asked the questions because they did not have any problem with the more challenging functional activities, we assigned a response of no difficulty. For Harmonized KLoSA IADL and ADL variables, not requiring help with the activity is counted as not having difficulty with the activity and needing help is counted as having difficulty.

Using these harmonized variables, we construct two disability indicators, one using IADLs and one using ADLs. IADL disability is defined as having difficulty with at least one of three IADLs. The three IADLs used for our analysis are managing money, taking medications, and shopping for groceries. ADL disability is defined as having difficulty with at least one of five ADLs. The five ADLs used for our analysis are bathing, dressing, eating, getting in or out of bed, and using the toilet.

### Statistical Analysis

Analyses in this paper were performed using Stata version 15. [34] In our analyses, we use the sampling weights provided by the surveys to ensure representativeness for the sampled population in each wave in each country. Disability prevalence for specific genders, countries, and years are presented as percentages. These percentages are estimated using weights which account for sampling design and potential bias from survey non- response. The percentage is the portion of population having the disability. Disability prevalence is compared between genders within a country at single time points and across time for each gender within a country. To test for differences in prevalence estimates between genders, Wald tests are used to test whether the estimates for men and women are statistically significantly different as reported with an F statistic. To test for differences across time, adjusted Wald tests are used to test whether the estimates at different time periods are statistically significantly different as also reported with an F statistic. Standard errors account for clustering at the individual level whenever data from multiple waves are combined. Country-level differences are inferred by presenting the mean, range, and ratio of the country, gender, time-specific disability prevalence estimates.

## Results

To study IADL and ADL disability prevalence we focused on two main comparisons: the cross-country variations in gender differences separately in 2004 and 2014 and the time trends for each gender and across countries. From these results, we find that of the 23 countries studied, only four countries in 2004 and only six in 2014 have significant gender differences in IADL disability; only two countries in 2004 and only five in 2014 have significant gender differences in ADL disability. We observe significant heterogeneity in time trends of IADL and ADL disability prevalence for both men and women in midlife, which are discussed in detail below.

### IADL Disability Prevalence

Table 2 displays population representative IADL disability prevalence among ages 55 to 65 by gender and country. There exist substantial cross-country variations in gender differences in IADL disability prevalence with the most disabled country having a prevalence at least seven times as high as the least disabled country for both males and female in both 2004 and 2014.

In 2004/05, the country with the highest IADL disability prevalence for men and women is England (7.5%, 9.2%). The country with the lowest IADL disability prevalence for men is Greece (0.4%) and for women is Czechia (2.2%). Women are estimated to have statistically significantly higher disability prevalence compared to men in Mexico (*p* < 0.001), Greece (*p* < 0.01), the United States (*p* < 0.01), and Belgium (*p* < 0.05).

In 2014/15 the country with the highest IADL disability prevalence for men is Portugal (10.3%) and for women is China (17.8%). The country with the lowest disability prevalence for men is Sweden (1.1%) and for women is Korea (0.8%). Women are estimated to have statistically significantly higher disability prevalence compared to men in China (*p* < 0.001), England (*p* < 0.001), Sweden (*p* < 0.001), Mexico (*p* < 0.01), and Germany (*p* < 0.05). Only in Korea are women estimated to have statistically significantly lower disability prevalence compared to men (*p* < 0.05).

These differences reflect different time trends in IADL disability prevalence by gender in different countries. IADL disability prevalence increased for both men and women in the U.S, but the increase was greater among men, resulting in no significant gender difference in IADL disability in the U.S. in 2014, when there had been a significant difference in 2004. In England, for men there was a significant decrease in their IADL disability prevalence over time while for women there was a small and non-significant change over the same time period, resulting in a significant gender gap in disability by 2014. IADL disability prevalence went up among men in Belgium, Czechia, and Greece, but not among women, leading to no observed gender difference in IADL disability in 2014. Among women, IADL disability prevalence went up in France but decreased in Korea, Poland, and Spain.

**Table 2:** IADL disability prevalence (in %) for ages 55-65 by gender and country

Country	2004/2005 Prevalence					2014/2015 Prevalence					Difference over Time		
	Men		Women		Difference	Men		Women		Difference	Men		`Women
	Estimate	SE	Estimate	SE		Estimate	SE	Estimate	SE				
Austria	2.0%	0.8%	3.6%	1.0%	1.6%	2.5%	0.8%	3.2%	0.9%	0.7%		0.5%	-0.5%
Belgium	2.4%	0.7%	4.9%	0.9%	2.5%*	4.4%	0.7%	4.9%	0.8%	0.5%		2.0%*	0.0%
China	.	.	.	.		9.7%	0.8%	17.8%	0.7%	8.1%***			
Croatia	.	.	.	.		1.8%	0.6%	3.3%	0.8%	1.5%			
Czechia	1.9%	0.6%	2.2%	0.6%	0.3%	3.9%	0.8%	3.3%	0.6%	-0.7%		2.1%*	1.1%
Denmark	2.7%	1.0%	4.9%	1.2%	2.2%	2.6%	0.7%	3.6%	0.7%	1.1%		-0.2%	-1.3%
England	7.5%	0.7%	9.2%	0.7%	1.7%	4.4%	0.7%	9.5%	1.0%	5.1%***		-3.1%***	0.3%
Estonia	.	.	.	.		5.0%	0.9%	4.9%	0.7%	0.0%			
France	2.7%	0.7%	2.2%	0.6%	-0.5%	3.8%	0.8%	5.0%	0.9%	1.2%		1.1%	2.7%**
Germany	3.2%	0.9%	3.9%	0.9%	0.8%	1.5%	0.5%	3.5%	0.7%	2.0%		-1.7%	-0.5%
Greece	0.4%	0.3%	2.9%	0.9%	2.5%**	1.7%	0.5%	3.0%	0.6%	1.2%		1.4%*	0.0%
Israel	4.8%	1.6%	7.7%	1.6%	2.8%	5.6%	3.1%	10.7%	2.4%	5.1%		0.8%	3.0%
Italy	3.9%	1.0%	3.1%	0.7%	-0.8%	2.6%	0.6%	3.4%	0.7%	0.8%		-1.3%	0.3%
Korea	3.2%	0.5%	3.1%	0.5%	-0.1%	2.1%	0.5%	0.8%	0.2%	-1.3%*		-1.1%	-2.3%***
Luxembourg	.	.	.	.		3.9%	1.2%	3.4%	1.0%	-0.6%			
Mexico	2.5%	0.5%	5.7%	0.7%	3.3%***	3.2%	0.6%	7.3%	1.1%	4.1%**		0.7%	1.6%
Poland	7.4%	1.3%	7.6%	1.2%	0.2%	7.7%	2.2%	3.4%	1.0%	-4.3%		0.3%	-4.2%**
Portugal	.	.	.	.		10.3%	6.3%	3.9%	1.1%	-6.4%			
Slovenia	.	.	.	.		2.8%	0.7%	2.4%	0.6%	-0.5%			
Spain	3.0%	1.3%	2.9%	0.8%	-0.1%	2.2%	0.7%	1.0%	0.4%	-1.1%		-0.8%	-1.9%*
Sweden	2.8%	0.8%	3.8%	0.8%	1.0%	1.1%	0.5%	5.9%	1.3%	4.8%***		-1.7%	2.1%
Switzerland	0.6%	0.6%	3.5%	1.4%	3.0%	1.4%	0.7%	1.8%	0.5%	0.4%		0.9%	-1.7%
USA	6.2%	0.6%	8.7%	0.6%	2.5%**	9.2%	0.6%	10.6%	0.6%	1.4%		2.9%***	1.9%*
Mean	3.4%		4.7%		1.3%	4.1%		5.1%		1.0%		0.2%	0.0%
Range	7.1		7.0		4.1	9.2		17.0		14.5		6.1	7.2
Ratio	19.7		4.2			9.6		21.5					

Notes: [1] Disability prevalence is the percentage of the population having an IADL disability at that time estimated using weights which account for sampling design and potential bias from with response rates.

[2] Disability is indicated by having difficulty with at least one of three instrumental activities of daily living (IADL) tasks: managing money, taking medications, and shopping for groceries.

[3] The range is the maximum value for an estimate minus the minimum value for an estimate, while the ratio is the maximum value for an estimate divided by the minimum value for an estimate.

[4] *indicates significance at the 0.05 level, ** indicates significance at the 0.01 level, *** indicates significance at the 0.001 level.

### ADL Disability Prevalence

Table 3 displays population-representative ADL disability prevalence among ages 55 to 65, by gender and country. There are substantial cross-country variations and gender differences in ADL disability prevalence with the most disabled country having a disability prevalence over 7 times as high as the least disabled country for both men and women in both 2004/05 and 2014/15. Cross-country variations are larger for women than men in both time-periods.

In 2004/05, the country with the highest ADL disability prevalence for men and women is Poland (17.1%, 16.7%) and the country with the lowest ADL disability prevalence for men and women is South Korea (2.4%, 1.5%). We observe gender differences in disability in only two out of 19 countries. Women are estimated to have statistically significantly higher disability prevalence compared to men in Greece (*p* < 0.05) and the United States (*p* < 0.01). In all other countries, there was no statistically significant gender difference in disability prevalence.

In 2014/15, the country with the highest ADL disability prevalence for both men and women is China (12.1%, 19.8%) and the lowest ADL disability prevalence for both men and women is South Korea (1.0%, 0.5%). Women are estimated to have statistically significantly higher disability prevalence compared to men in China (*p* < 0.001), Mexico (p < 0.001), and England (*p* < 0.05). In Czechia and Denmark women estimated to have statistically significantly lower disability prevalence compared to men (*p* < 0.05 in both countries).

We observe different time trends (or cohort differences) when comparing 2004/2005 with 2014/2015 ADL prevalence. In Czechia, ADL disability prevalence increased by 7% among men, but remained stable among women, which contributed to a female advantage in terms of ADL prevalence. Contrastingly, in England the ADL disability prevalence decreased by 4.7% for men but remained relatively stable for women, contributing to gender difference in disability prevalence in favor of men. Women in Mexico experienced 6.5% increase in ADL disability prevalence, which lead to a gender difference in ADL disability by 2014/15. ADL prevalence decreased during this time-period among both men and women in Korea and Poland, but no gender differences are observed in these two countries in 2014/2015. ADL disability decreased for Danish women over this time period, and in 2014/15 Danish women had a lower disability prevalence than Danish men. In France, ADL prevalence increased for women but did not result in a gender difference in 2014/2015.

**Table 3:** ADL disability prevalence (in %) for ages 55-65 by gender and country

Country	2004/2005 Prevalence					2014/2015 Prevalence					Difference over Time	
	Men		Women		Difference	Men		Women		Difference	Men	Women
	Estimate	SE	Estimate	SE		Estimate	SE	Estimate	SE			
Austria	6.1%	1.5%	6.6%	1.3%	0.5%	5.0%	1.3%	4.9%	1.1%	-0.1%	-1.1%	-1.7%
Belgium	7.5%	1.1%	8.1%	1.1%	0.6%	9.0%	1.2%	8.2%	0.9%	-0.8%	1.5%	0.1%
China	.	.	.	.		12.1%	0.6%	19.8%	0.7%	7.8%***		
Croatia	.	.	.	.		6.1%	1.2%	7.3%	1.1%	1.2%		
Czechia	4.0%	1.1%	5.3%	1.2%	1.3%	11.0%	1.9%	5.9%	0.8%	-5.1%*	7.0%**	0.6%
Denmark	6.9%	1.5%	6.8%	1.4%	0.0%	6.1%	1.0%	3.4%	0.7%	-2.7%*	-0.8%	-3.4%*
England	15.3%	0.9%	15.0%	0.8%	-0.4%	10.6%	1.0%	13.8%	1.1%	3.2%*	-4.7%***	-1.1%
Estonia	.	.	.	.		9.5%	1.2%	8.4%	0.9%	-1.1%		
France	6.9%	1.2%	5.6%	1.0%	-1.3%	6.3%	1.0%	8.7%	1.1%	2.4%	-0.6%	3.1%*
Germany	6.3%	1.2%	7.3%	1.2%	1.0%	6.9%	1.1%	6.8%	1.0%	-0.1%	0.6%	-0.5%
Greece	2.6%	0.8%	5.6%	1.2%	3.0%*	2.4%	0.6%	4.2%	0.7%	1.8%	-0.2%	-1.5%
Israel	8.2%	1.9%	10.6%	2.1%	2.3%	4.1%	1.2%	5.3%	1.8%	1.3%	-4.1%	-5.2%
Italy	5.1%	1.2%	5.7%	1.0%	0.6%	4.5%	0.8%	4.2%	0.7%	-0.3%	-0.6%	-1.4%
Korea	2.4%	0.4%	1.5%	0.3%	-0.9%	1.0%	0.3%	0.5%	0.2%	-0.5%	-1.4%**	-1.0%**
Luxembourg	.	.	.	.		4.8%	1.2%	8.0%	1.5%	3.2%		
Mexico	4.8%	0.7%	6.2%	0.6%	1.3%	7.1%	0.9%	12.7%	1.3%	5.6%***	2.3%	6.5%***
Poland	17.1%	1.9%	16.7%	1.7%	-0.4%	10.2%	1.9%	7.9%	1.4%	-2.2%	-6.9%*	-8.7%***
Portugal	.	.	.	.		11.6%	5.7%	16.6%	3.8%	5.0%		
Slovenia	.	.	.	.		7.1%	1.0%	6.1%	0.9%	-0.9%		
Spain	5.9%	1.5%	6.7%	1.2%	0.8%	4.5%	1.4%	5.7%	1.7%	1.1%	-1.4%	-1.0%
Sweden	5.4%	1.0%	5.6%	1.0%	0.2%	4.2%	1.0%	7.3%	1.4%	3.1%	-1.2%	1.7%
Switzerland	3.8%	1.5%	2.4%	1.2%	-1.4%	3.7%	1.0%	3.5%	0.8%	-0.3%	-0.1%	1.1%
USA	9.8%	0.7%	12.9%	0.7%	3.1%**	11.3%	0.7%	12.2%	0.6%	0.9%	1.5%	-0.7%
Mean	6.9%		7.5%		0.6%	6.9%		7.9%		1.0%	-0.6%	-0.8%
Range	14.7		15.1		4.5	11.0		19.3		12.9	13.9	15.3
Ratio	7.1		10.8			11.8		37.4				

Notes: [1] Disability prevalence is the percentage of the population having an ADL disability at that time estimated using weights which account for sampling design and potential bias from with response rates.

[2] Disability is indicated by having difficulty with at least one of five activities of daily living (ADL) tasks: bathing, dressing, eating, getting in and out of bed, and using the toilet.

[3] The range is the maximum value for an estimate minus the minimum value for an estimate, while the ratio is the maximum value for an estimate divided by the minimum value for an estimate.

[4] *indicates significance at the 0.05 level, ** indicates significance at the 0.01 level, *** indicates significance at the 0.001 level.

## Discussion

Our study examines mid-life gender differences of disability prevalence measured using both IADLs and ADLs across 23 countries and two time periods. We find substantial variations in disability prevalence in midlife across countries, however in most countries, we observe no gender difference in disability in midlife adults. This a significant expansion to the current literature which has generally either only focused on a single country or pooled countries together. In comparison to earlier literature we find gender differences in disability even in countries which are often considered similar. For example, we find an IADL gender difference in 2004//05 in Belgium but not in France. This suggests that pooling countries together could hide country-level differences in gender disparities in disability.

The cross-country variability in disability prevalence might be driven by various social determinants of health, including education, employment, food security, housing, and economic status [35]. These factors may vary drastically across countries, causing different patterns in gender differences in disability. For example, we observe a female advantage in 2014/15 in IADL disability in Korea. This observed difference could be related to culturally specific gender roles in Korea relating to shopping for groceries which may lead to an unmet care need among men.

Our study also has the advantage of considering two time points which allows us to examine time or cohort trends and better compare our findings to previous literature. In contrast to Scheel-Hincke et al., [25] we find gender differences in IADL disability changing over time in six European countries, and gender differences in ADL disability changing over time in four European countries. Expanding on earlier literature which measured disability in midlife between 2001 and 2005, when examining gender disparities in 2014/15, we find that the disparity has decreased in the US but has increased in England and Mexico compared to 2004/05.

Overall, we observe different time trends in both IADL and ADL disability across countries. Specifically for IADL disability, we observe decreasing prevalence in four countries and increasing prevalence in five countries. IADL disability decreased for women in Korea, Poland, and Spain. Declining prevalence of IADL disability may be due to the improvement of socioeconomic and living environments in rural areas [36]. Furthermore, since IADLs require more complex cognitive ability, increased education levels in younger cohorts may contribute to declining prevalence of IADL disability [37]. The observed cross-country variation may be explained by differences in progress and presence of educational equality across different educational systems [38]. We also observe increasing IADL disability prevalence among women in France and the United States. The reason for such trend is not clear, but a potential explanation could be poor economic conditions. Choi found an increase in IADL difficulty between 2008 and 2012 in the United States and contributed this increase to the Great Recession and economic difficulties which prevent people from purchasing medications and food [39].

In comparison to time trends in IADL disability we find slightly fewer time trends in ADL disability prevalence. We find that ADL prevalence decreased in four countries and increased in two. Specifically for women, we find that ADL disability decreased in Denmark, Korea, and Poland and ADL disability increased in France and Mexico. The decreasing prevalence may be due to the improvement in population health and living environments or increase use of aids or technological devices that are facilitating greater independence for older adults. Furthermore, improvements in medical and health services lead to preventative diagnoses and better treatments of diseases and disabilities [40]. The increased prevalence we observed could be a result of increased health-related conditions like diabetes over the same time span [41].

From our results, we see that the patterns of disability change over time are not are not consistent between countries or genders. The US is the only country that has a significant increase for both men and women in IADL disability in midlife over time. Poland and Korea are the only countries that show consistent ADL disability prevalence decreasing trends over time for men and women. These variations suggest that disability is a much more dynamic and multifaceted issue that requires further understanding and analysis.

Our study separately examined IADL and ADL disability prevalence and found different gender disparities and time trends in IADL and ADL disability. Previous research on gender differences in disability at midlife have used a number of different measures to identify disability including composite measures of health and functioning, mobility limitations, and combined IADL and ADL difficulties. We observe a general lower prevalence of IADL than ADL disability across countries, aligning with other current literature focusing on midlife disability [42]. However, among older adults, the prevalence of IADL impairment typically is larger than that of ADL impairment [43–46]. In older adults, the higher IADL disability prevalence is thought to reflect a hierarchical disabling process. This process explains that the loss of independence in IADLs comes before that of ADLs due to the cognitive impairments in older adults that may affect the ability to perform more cognitively complex IADL tasks [47, 48]. Thus, the observed lower burden of IADL disability in mid-life may reflect a lower prevalence of cognitive impairment and neurodegenerative diseases in our middle-aged cohort.

A strength of this study is the rich longitudinal data used. We were able to examine 10-year time trends in IADL and ADL disability prevalence in 19 countries. The data used have adequate statistical power and are from nationally representative samples. Furthermore, the data was collected with the aim of cross-country comparability. However, some limitations should be noted. Even though data were collected with the purpose of cross-country comparability there are differences by study in answers choices, survey skip pattern, and question wording. Many of these differences were addressed in the creation of the harmonized variables but the degree to which harmonization was not able to account for all the differences could affect their cross-country comparability. Because the analysis mainly focuses on differences between genders in the same country and differences over time in the same country, we do not expect much bias to result from the cross-study differences as these questions did not change over time. But when comparing disability prevalence between countries these differences become more important and could be misleading. For instance, we observe very low IADL and ADL disability prevalence in Korea where respondents were asked whether they needed help with the activity and not whether they had difficulty with the activity, as was asked in all other countries. Even though a large number of countries were compared, many low-income countries were not included due to data unavailability. Additionally, there are comparable studies in other countries which did not survey all the activities we used for our ADL and IADL summary, including Costa Rica and Japan, so they could not be included in our analysis.

Despite these limitations, this study is one of the first to provide empirical evidence of gender differences in disability prevalence across as many as 23 countries. Furthermore, we examine analyze time trends in disability in 19 of those countries. This study is one of the first cross-country studies to focus specifically on gender differences in midlife adults. As this study finds some countries fare better than others in disability prevalence among men and women, it is important to conduct further research to identify what factors contributes to these cross-country differences. This would include better understanding the intersectionality between country and gender differences in disability due to economic status, co-morbidities, health behaviors, biology and physiology, and other potentially confounding factors. Moreover, this study finds that certain countries have seen a decrease in disability prevalence for women between 2004/05 and 2014/15, calling for further investigation into important lessons for the global community. Utilizing internationally harmonized data, there is a unique opportunity to better understand aging and the factors that may contribute to the cross-country variations in the aging process on a global scale.

## Conclusions

This study focused on gender differences in disability during midlife across 23 different countries and in two time periods. We observed significant cross-country variations in gender differences in disability prevalence in midlife and varying trends over time. It is important to note that on average, we do not observe any statistically significant gender difference nor time trend in midlife disability globally, but at country-level, significant heterogeneity exists both in terms of gender difference and time trend in midlife. This pattern calls for further study into the factors that cause these cross-country differences. It also presents a need for interventions to reduce disability at an early life stage in countries before higher prevalence of disability is manifested. Understanding the global context of this health disparity further highlights the opportunity for further research in these areas and the potential benefits of interventions.

## Data Availability

All data are public and can be accessed via the Gateway to Global Aging Data (g2aging.org).

## Abbreviations

ADL: Activities of daily living
IADL: Instrumental activities of daily living
Gateway: Gateway to Global Aging Data
HRS: Health and Retirement Study
MHAS: Mexican Health and Aging Study
ELSA: English Longitudinal Study of Ageing
KLoSA: Korean Longitudinal Study of Aging
SHARE: Survey of Health, Ageing, and Retirement in Europe
CHARLS: Chinese Health and Retirement Longitudinal Study

## Footnotes

1 Northern Europe included Denmark and Sweden; Western Europe included Austria, Germany, France, the Netherlands, Switzerland, Belgium, Ireland and Luxembourg; Southern Europe included Spain, Italy, Greece and Portugal; and Eastern Europe included Czech Republic, Poland, Hungary, Slovenia, Estonia and Croatia.

2 There are a few exceptions to the questionnaire frequency, respondent age, and spouse inclusion for these surveys. Even though the majority of surveys conduct interviews biennially, the MHAS experienced a 9-year interval between their 2003 and 2015 wave. MHAS, ELSA, and SHARE interview respondents aged 50 and older, the HRS interviews respondents 51 and older, KLoSA and CHARLS interview respondents aged 45 and older. KLoSA is the only survey that interviews only age-eligible spouses while others include spouses regardless of age. It is also important to note the number of countries included in SHARE varies across waves. Our analysis includes all SHARE countries which participated in their wave 6, conducted in 2015.
